# Exploring neonicotinoid effects on *Drosophila*: insights into olfactory memory, neurotransmission, and synaptic connectivity

**DOI:** 10.3389/fphys.2024.1363943

**Published:** 2024-03-14

**Authors:** Julia Schulz, Hanna R. Franz, Stephan H. Deimel, Annekathrin Widmann

**Affiliations:** Department of Molecular Neurobiology of Behavior, University of Göttingen, Göttingen, Germany

**Keywords:** neonicotinoids, imidacloprid, *Drosophila melanogaster*, olfactory memory, cholinergic neurotransmission, mushroom body network

## Abstract

Neonicotinoid insecticides, the fastest-growing class in recent decades, interfere with cholinergic neurotransmission by binding to the nicotinic acetylcholine receptor. This disruption affects both targeted and non-targeted insects, impairing cognitive functions such as olfaction and related behaviors, with a particular emphasis on olfactory memory due to its ecological impact. Despite the persistent presence of these chemicals in the environment, significant research gaps remain in understanding the intricate interplay between cognitive function, development, neuronal activity, and neonicotinoid-induced toxicity. This study focuses on the fruit fly *Drosophila melanogaster*, chosen for its genetic tractability, well-characterized neural circuitry, and remarkable parallels with bees in neurotransmitter systems and brain structures. Our aim is to establish the fruit fly as a valuable model organism for studying the effects of neonicotinoids on behavior and neuronal circuitry, with particular attention to olfactory memory and associated brain circuitries. To achieve this aim, we conducted experiments to investigate the effects of short-term exposure to sublethal doses of the neonicotinoid imidacloprid, mimicking realistic environmental insecticide exposure, on the formation of odor memories. Additionally, we evaluated synaptic contacts and cholinergic neurotransmission within the mushroom body, the primary memory network of insects. Our results showed significant impairments in odor memory formation in flies exposed to imidacloprid, with exposure during the adult stage showing more pronounced effects than exposure during the larval stage. Additionally, functional studies revealed a decrease in synaptic contacts within the intrinsic olfactory projection neurons and the mushroom body. Furthermore, another experiment showed an odor-dependent reduction in cholinergic neurotransmission within this network. In summary, employing *Drosophila* as a model organism provides a robust framework for investigating neonicotinoid effects and understanding their diverse impacts on insect physiology and behavior. Our study initiates the establishment of the fruit fly as a pivotal model for exploring neonicotinoid influences, shedding light on their effects on olfactory memory, neuronal integrity, and synaptic transmission.

## 1 Introduction

Pollinating insects are essential for the overall health and balance of an ecosystem and are under increasing threat from widespread pesticide use (Vanbergen and Initiative, 2013). Neonicotinoids, in particular, have been shown to entail adverse effects on non-target organisms, especially pollinators, despite their high specificity and efficacy against target species. Studies have shown that neonicotinoids not only increase mortality in bees (Goulson, 2013), but also have sublethal effects on various bee species at environmentally relevant doses. For example, exposure to sublethal doses of neonicotinoids during the adult stage affects behaviors such as learning and memory (Aliouane et al., 2009; Williamson and Wright, 2013; Samuelson et al., 2016), locomotion and motor function (Lambin et al., 2001; Tomé et al., 2012; Cresswell et al., 2014; Williamson et al., 2014; Tosi et al., 2017; Tosi and Nieh, 2017; Alkassab and Kirchner, 2018; Jacob et al., 2019), foraging activity and motivation (Henry et al., 2012; Schneider et al., 2012; Gill and Raine, 2014; Lämsä et al., 2018; Tasman et al., 2020), grooming (Morfin et al., 2019), and circadian rhythms and sleep (Tackenberg et al., 2020; Tasman et al., 2020). At the same time, these pesticides reduce the fertility of drones and queens, negatively affecting reproductive success and colony growth (Whitehorn et al., 2012; Straub et al., 2016; Wu-Smart and Spivak, 2016; Crall et al., 2018).

Despite the persistence of neonicotinoids in the environment (Botías et al., 2015) and the recognized effects of exposure on individuals at the adult stage, a notable research gap exists regarding the impacts of neonicotinoid exposure at different developmental stages. For holometabolous insects like bees, the transition from the larval stage, which is characterized by prolonged active feeding and limited mobility, to the adult stage characterized by increased mobility and reproductive capacity, is critical (Rolff et al., 2019). Due to the distinct physiologies and functions associated with each phase of life, it is imperative to thoroughly investigate the effects of neonicotinoid exposure at different developmental stages. To this end, comparative studies are essential for a nuanced understanding of how neonicotinoids influence insect cognitive functions and neuronal structuring of the brain throughout the entire life cycle. The urgency for more extensive investigations is underscored by the existence of only limited studies on larval neonicotinoid exposure and its impacts on olfactory learning in bees (Yang et al., 2012; Tan et al., 2015; Tan et al., 2017; Peng and Yang, 2016).

Our study focused on the fruit fly *Drosophila melanogaster*, offering a distinctive opportunity to evaluate the impacts of neonicotinoid exposure across various dimensions, including different behaviors and brain circuitries. The fruit fly’s relatively small neural network of approximately 100,000 neurons has been extensively studied, including the complete connectome of the brain (Eichler et al., 2017; Takemura et al., 2017; Zheng et al., 2018; Raji and Potter, 2021). *Drosophila* and bees share genetic similarities in neurotransmitters and receptors—particularly nicotinic acetylcholine receptors (nAChRs), which are the primary targets of neonicotinoids (Hauser et al., 2006; The Honeybee Genome Sequencing Consortium, 2006; Velarde et al., 2006; Jones and Sattelle, 2010; Blenau and Thamm, 2011; Dupuis et al., 2012; Matsuda et al., 2020). Both fruit flies and bees possess nAChRs in brain regions responsible for olfactory processing and learning and memory (Jones and Sattelle, 2010). In addition, neonicotinoids have been shown to impact reproduction, lifespan, motor function, learning and memory, and circadian rhythm in fruit flies (Charpentier et al., 2014; Li et al., 2020; Tasman et al., 2021b). Therefore, drawing parallels between fruit flies and bees in terms of genetic similarities, neurotransmitter systems and brain structure allows for a more nuanced understanding of the interplay between insecticide toxicity and insect physiology.

Our study aimed to investigate the effects of realistic environmental exposure to neonicotinoids on behavior and neuronal circuitry in *Drosophila*, specifically focusing on olfactory memory and associated brain circuitries. To achieve this, we conducted experiments to assess the impact of short-term exposure to sublethal doses of imidacloprid, a common neonicotinoid insecticide, on odor memory formation and synaptic transmission within the primary memory network of insects, known as the mushroom body (MB). This network involves MB Kenyon cells (KCs), which play a crucial role in olfactory processes by receiving cholinergic input from projection neurons (PNs) onto the MB calyx (Widmann et al., 2018; Davis, 2023; Thum and Geber, 2019). Our results demonstrated notable impairments in odor memory formation among flies exposed to imidacloprid. Our findings suggest differential impacts on different memory components, highlighting the nuanced effects of varying concentrations of imidacloprid. Interestingly, we observed varying degrees of severity in these impairments based on the developmental stage of exposure; adult-stage exposure exhibited more pronounced effects compared to larval-stage exposure. Additionally, functional studies demonstrated a decrease in synaptic contacts within the intrinsic olfactory PNs and the MB, accompanied by an odor-dependent reduction in cholinergic neurotransmission within this network.

Overall, our study highlights differential impacts on various memory components and emphasizes the importance of considering developmental stages when assessing neonicotinoid effects on insect physiology. Furthermore, it initiates the exploration of the fruit fly brain as a pivotal model for investigating neonicotinoid influences, shedding light on their effects on olfactory memory, neuronal integrity, and synaptic transmission.

## 2 Material and methods

### 2.1 Fly stocks

Fly strains were reared on standard *Drosophila* medium at 25°C with 60% humidity under a 12-h light-dark cycle, unless indicated otherwise. The wild-type strain Canton-S (referred to here as wild-type) was used for all behavioral experiments. For pupation and eclosion assay the strain *w* ^*1118*^ was used. To analyze synaptic connectivity between PNs and the MB, the fly strain *w* ^*-*^ *;mb247-DsRed;mb247-splitGFP11,UAS-splitGFP1-10* (MB-splitGFP) (Pech et al., 2013) was crossed with *GH146*-Gal4 (Stocker et al., 1997) to obtain reconstituted splitGFP-dependent fluorescence in this area. To measure cholinergic activity in the MB *in vivo*, we used the optimized genetically encoded GPCR activation based acetylcholine sensor *UAS-GACh3.0* (BDSC#86549) (Jing et al., 2020) combined with *w* ^*1118*^ *;;mb247:mCherry-CAAX* (III) (Kobler et al., 2021) and the KC-specific line *mb247*-Gal4/*CyO* (referred to here as *mb247*-Gal4) (gift from Elisabeth J. Hong). Stocks used in this study are listed in S2 Table.

### 2.2 Imidacloprid treatment

**FIGURE 1:**
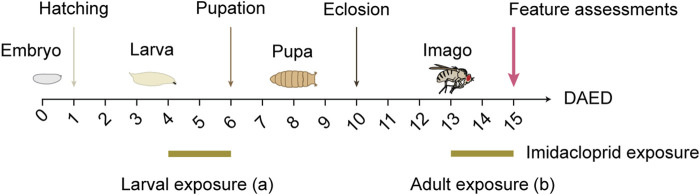
Schematic illustration of larval or adult exposure to imidacloprid. *Drosophila* were maintained at 25°C on a 12/12 h day/night cycle. For larval imidacloprid exposure (a), third-instar larvae were exposed to imidacloprid for 2 days starting at 4 days after egg deposition (DAED). For adult imidacloprid exposure (b), 3-day-old adult flies (13 DAED) were exposed to imidacloprid for 2 days. Behavioral or morphological characteristics were then examined in 5-day-old flies (15 DAED).

To study the short-term effects of imidacloprid in *Drosophila*, third-instar larvae and adult flies underwent a 2-day treatment (Figure 1). Larvae were treated from 4 days after egg deposition (DAED) to avoid molting interference. Adult flies, aged 3 days (13 DAED), were exposed for 2 days to allow uninterrupted brain maturation. Behavioral and morphological assessments were conducted in 5-day-old flies (15 DAED). Due to its low solubility in water (Kong et al., 2008), imidacloprid (Sigma-Aldrich, Cat#37894) was dissolved in 0.001% v/v dimethyl sulphoxide (DMSO, Sigma-Aldrich, Cat#D2650), which is part of the commercial formulation (Costa et al., 2009), and then stored at room temperature. To exclude possible DMSO-related effect, the amount of DMSO in the imidacloprid solution was below 0.001%, v/v. Control animals received DMSO at a 0.001% concentration as a vehicle treatment. To ensure that no traces of imidacloprid remained in the pupa during metamorphosis, larvae were carefully rinsed with autoclaved tap water and then transferred to standard fly food.

### 2.3 Pupation and eclosion assay

To determine the imidacloprid concentration corresponding to sublethal levels, a pupation and eclosion assay was conducted. Here, *w* ^*1118*^ larvae were exposed to varying imidacloprid concentrations in standard cornmeal food at 4 days after egg deposition (DAED). After a 2-day exposure, larvae were carefully rinsed and individually transferred to custom-made wells with a diameter of 9 mm in 50-well plates. The wells were filled with 200 μL of apple-juice-yeast food, sealed with an air-permeable lid, and incubated in slightly opened plastic containers. To maintain a constant humidity, paper towels at the bottom of the containers were misted with water daily. Throughout a 7-day observation period, this setup was placed in a 25°C incubator with 60% humidity.

### 2.4 Naïve odor perception

To test the flies’ sense of smell, groups of 30–60 animals were tested in a T-maze, with the temperature and relative humidity set at 25°C and 60%–80%, respectively. Before the start of each experiment, the flies were placed in empty containers and left for 1 min. The animals were then given access to a two-armed selection point consisting of two tubes containing either the respective odor or pure mineral oil. The flies were allowed to move freely for 2 min and choose between the tube containing the odor and the tube containing the mineral oil. After the odor exposure, the number of animals in each tube was counted and an odor perception index was calculated as the difference between the number of flies on the odor side and on the mineral oil side, divided by the total number of flies on both sides. Negative scores indicate odor avoidance, and positive scores indicate odor preference.

### 2.5 Naïve shock perception

To assess the shock sensitivity of flies, groups of 30–60 animals were tested in a T-maze assay with temperature and relative humidity adjusted to 25°C and 60%–80%, respectively. Prior to the start of each experiment, flies were transferred to empty vials and left for 1 min. The flies were then given access to a two-armed choice point, which consisted of two opposing tubes internally covered with an electrifiable grid, only one of which was connected to the voltage source and delivered an electric shock. Shocks were delivered at a frequency of one every 5 s for 1.25 s, with 90 V pulses lasting 2 min, for a total of 24 shocks. The flies were allowed to move around freely for 2 min and to choose between the tube that had delivered an electric shock and the tube that had not. After the shock exposure, the number of animals in each tube was counted and an odor perception index was calculated as the difference between the number of flies on the odor side and on the mineral oil side, divided by the total number of flies on both sides. Negative scores indicate avoidance, and positive scores indicate preference.

### 2.6 Aversive olfactory learning and memory

Aversive olfactory learning was performed at 25°C and a relative humidity of 60%–80% under standard laboratory conditions, as previously described (Tully and Quinn, 1985), but with some modifications. Briefly, groups of 30–60 animals were trained and tested in a T-maze assay. 4-methylcyclohexanol (MCH, Sigma-Aldrich, Cat#153095) and 3-octanol (3-OCT, Sigma-Aldrich, Cat#218405) diluted in mineral oil (Sigma-Aldrich, Cat#M8410) were used as olfactory stimuli. Flies were placed in tubes covered inside with an electrifiable grid and the training started 1 min later. Each odor was presented for 1 min, with a 1 min break between the two odor applications. One odor was temporally paired with 12 electric shocks of 90 V DC (1.25-s shock duration and 3.75-s inter-pulse interval) (conditioned stimulus +, CS+), whereas the second odor was presented without shock (conditioned stimulus -, CS-). Learning and memory was tested by transferring flies to the T-maze part of the apparatus with both odors presented on opposite sites. After 2 min the animals on each side were counted, and a preference index was calculated by subtracting the number of animals on the side associated with the CS- from the number of animals associated with the CS+, divided by the total number of animals. To specifically measure the effect of associative learning, we then calculated a learning index by averaging preference indices from two reciprocal experiments. Negative scores indicated aversive olfactory learning, and positive scores indicated appetitive olfactory learning. For the assessment of 3-min short-term memory, we employed a single-training paradigm (1x training) and tested the memory 3 min after training. Conversely, to examine 24-h long-term memory, we utilized a spaced training paradigm involving five trials with 15-min intervals (5x spaced training) and tested the memory 24 h after training (Tully and Quinn, 1985; Tully et al., 1994).

### 2.7 Locomotor activity using a DAM2 *Drosophila* activity monitor

To monitor adult *Drosophila* locomotion, individual male wild-type flies were placed in glass tubes (5-mm diameter, 6.5-cm length), with one end filled with fly food and sealed with parafilm and the other with an air-permeable plug. Locomotion was then monitored by placing the glass tubes in the DAM2 *Drosophila* Activity Monitor (TriKinetics Inc.). The DAM2 recorder was kept in a humidity and temperature-controlled incubator under a 12-h light/dark cycle for 3 consecutive days. Data were analyzed using ShinyR-DAM software from Cichewicz and Hirsh (2018).

### 2.8 Immunohistochemistry

The brains were dissected in Ringer’s solution and washed with phosphate-buffered saline (PBS) before fixation in a solution of 4% paraformaldehyde (PFA). After fixation, the brains underwent three washes with PBS containing 0.6% Triton X-100. Blocking was performed using a solution containing 2% normal goat serum (NGS), 2% bovine serum albumin (BSA), and 96% PBST overnight at 4°C. Subsequently, the brains were incubated with primary antibodies (a-DLG, 1:50; a-GFP, 1:1000) for 6 hours at room temperature. After three washes with PBST, secondary antibodies (a-mouse Alexa 488, 1:300; a-chicken Alexa 633, 1:300) were applied overnight at 4°C. Following three washes with PBST, the brains were mounted in Vectashield and image stacks were obtained a Leica TC SP8 confocal microscope and a 20x/0.7 water/glycerol immersion objective at 1-µm steps in the *z*-axis.

### 2.9 Quantification of reconstituted splitGFP

For immunohistochemistry, flies were anesthetized on ice before dissecting the brains in Ringer’s solution. The fixation was performed in 4% paraformaldehyde (PFA) dissolved in phosphate-buffered saline (PBS) for 30 min at room temperature. Subsequently, the samples were washed three times with PBS containing 0.6% Triton-X 100 for 20 min each. Finally, the brains were mounted into a 6-µL drop of VECTASHIELD Mounting Medium (Vector laboratories, Cat#H-1000) within a reinforcement ring on a microscope slide. Imaging was conducted within 48 h after mounting employing a Leica TC SP8 confocal laser scanning microscope equipped with a Leica Apochromat ×20 air objective (×20 HC PL Apo CS, NA = 0.70). Reconstituted splitGFP (rsGFP) was excited at 488 nm and DsRed, which serves as a background marker within the mushroom body (MB), at 561 nm wavelengths. The MB region of each brain was scanned at 1-μm sections in the *z*-direction with a zoom factor of 3x and a resolution of 5.28 pixel/μm. All imaging settings such as laser excitation power, fluorophore detection ranges, zoom factor, and z-step size were constant between experiments. To quantify rsGFP between PNs and KCs, we employed Fiji software (Schindelin et al., 2012) along with the 3D ImageJ Suite plugin (Ollion et al., 2013). The precise calyx region was determined individually for each MB and z-stacks were cut accordingly. The DsRed and rsGFP signals of each z-stack were then subjected to filtering using the 3D fast filter (median, radius x, y, z = 2 pixels, parallelized algorithm, Nb cpus = 8). The DsRed signal was utilized to create a binary mask for automatic regions of interest (ROI) assignment in each brain. This involved segmenting the DsRed channel using 3D hysteresis thresholding (high threshold: 40, low threshold: 10) and smoothing the edges using the 3D binary close labels function (x, y = 5 pixels, z = 3 pixels, no dilation). The resulting binary mask was loaded into the 3D ROI manager and employed to quantify the 3D pixel intensities (integrated densities; IntDen) of the DsRed and rsGFP signals within the respective calyx. The resulting rsGFP integrated density was divided by the integrated density of the DsRed channel to correct for general changes in fluorescent signal strength between brains [see Eq. (1)]. Subsequently, the corrected integrated density value was normalized to the mean of the corrected integrated density of the DMSO control group [see Eq. (2)].

$${IntDen}_{corrected}=IntDen\left(rsGFP\right)\;/\;IntDen\left(DsRed\right)$$ (1)

$${IntDen}_{normalized}=\left({IntDen}_{corrected}-\bar{\left.{IntDen}_{corrected}\left(DMSO\right)\right)}/\bar{{IntDen}_{corrected}\left(DMSO\right)}\right.$$ (2)

### 2.10 Two-photon imaging in *Drosophila*

For *in vivo* functional imaging, adult female flies were briefly anaesthetized on ice, mounted into a custom-built fly chamber and the dorsal head capsule was opened and covered with Ringer’s solution (Hancock et al., 2019). Imaging was performed at a framerate of 4 Hz with a 2-photon laser-scanning microscope LSM 7 MP (Zeiss) equipped with a W Plan-Apochromat 20x/1.0 DIC M27 75 mm objective (Zeiss). Here, the GACh3.0 sensor and mCherry, which serves as morphological marker for the calyx, were both excited at 920 nm using a Ti:sapphire laser (Coherent Chameleon). To enable simultaneous detection of their emitted fluorescence signals, a dichroic mirror and bandpass filters (500–550 nm for GACh3.0 and 650–660 nm for mCherry) were used. The odors MCH, 3-OCT, and BEN (Sigma-Aldrich, Cat#B1334) were administered using a custom-built olfactometer, with each odor presented sequentially for 3 s, separated by 40-s intervals in which only mineral oil was administered. The two-photon images obtained were analyzed using Fiji software (Schindelin et al., 2012). To correct for motion, the raw image sequences were processed using the TurboReg plugin in ImageJ (Thevenaz et al., 1998). A single ROI of the entire calyx region was determined by creating a maximum z-projection, and mean intensity values (F) over the time course of 25 s with discrete time step intervals of 0.25 s were calculated. To calculate ΔF/F0, F0 was defined as the mean value during the 5-s period before the onset of the odor. To statistically compare responses, each was quantified by calculating the area under the curve (AUC) of the ΔF/F0 time series. This was done by integrating the signal from the onset of the odor stimulus to 3 s after its offset, covering the time interval from 6.25 s to 12.25 s.

### 2.11 Quantification and statistical analysis

All statistical analyses and visualizations were performed using GraphPad Prism 9, with a significance level set at α = 0.05. We evaluated whether the responses of groups were significantly different from chance using Holm-Sidak-corrected, two-tailed, one-sample *t* tests for normally distributed data (as determined by the Shapiro-Wilk test), and Holm-Sidak-corrected two-tailed Wilcoxon signed-rank tests for non-normally distributed data. We used parametric statistics to statistically analyze differences between groups that met parametric assumptions (i.e., normal distribution, as determined by the Shapiro-Wilk test, and homogeneity of variance, as determined by the Bartlett’s test). Specifically, an unpaired *t*-test or Mann-Whitney test was used for comparisons between two groups. Two compare against the DMSO-treated control group for more than two groups, either a one-way ANOVA followed by Dunnett’s multiple comparison or a Kruskal–Wallis followed by Dunn’s multiple pairwise comparison was conducted. Detailed information about the specific statistical tests used, sample sizes, and descriptive statistics can be found in Supplementary Tables S3–S5 for the main figures and the Supporting Information figures. Figure alignments were performed using Adobe Illustrator CC 2022.

## 3 Results

### 3.1 Concentration selection for short-term imidacloprid exposure in larval and adult *Drosophila*

**FIGURE 2:**
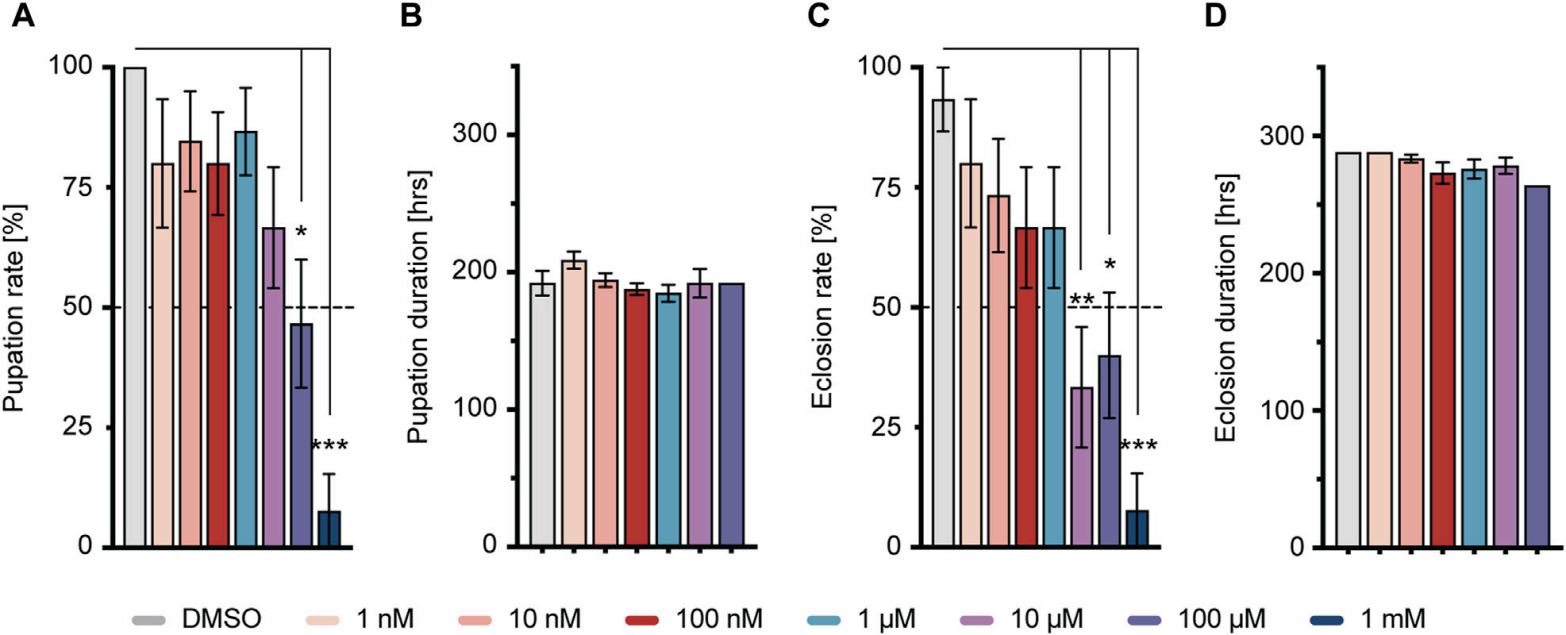
Larval response to imidacloprid and determination of sublethal exposure concentration. **(A)** Pupation time was not significantly affected by different concentrations of imidacloprid. **(B)** Larvae treated with imidacloprid at concentrations of 100 μM and 1 mM showed a significant reduction in pupation percentage compared to the DMSO-treated group. **(C)** The eclosion time of adult flies was not significantly affected by different concentrations of imidacloprid. **(D)** Larvae treated with imidacloprid at concentrations of 10 μM, 100 μM, and 1 mM showed a significant reduction in eclosion percentage compared to the DMSO-treated group. Data are presented as bar graphs with mean values and SEM represented by error bars. Statistical significance compared to the DMSO-treated group is indicated above the graph bars (***p < 0.001, **p < 0.01, *p < 0.05). See Supplementary Tables S3, S5 for further statistical details.

To examine the impact of short-term imidacloprid exposure on both larval and adult stages of *Drosophila*, third-instar larvae, 4 days after egg deposition (DAED) or 3-day-old adult flies (13 DAED) underwent a 2-day exposure to sublethal concentrations of imidacloprid. We then conducted a comprehensive evaluation of various features including olfaction, memory, synaptic connectivity, and cholinergic neurotransmission in 5-day-old flies (15 DAED) (Figure 1). Given the documented adverse effects of concentrations exceeding 50 μM on adults (Tatarko et al., 2023), our initial analysis focused on assessing the comparative effects during the larval stage, with a specific emphasis on successful pupation and eclosion. This approach aimed to ensure consistency in concentration usage across larvae and adults. *w* ^*1118*^ larvae exposed to varying concentrations of imidacloprid at 4 days after egg deposition (DAED) exhibited a significant, dose-dependent reduction in pupation percentage at concentrations of 100 μM and 1 mM, and in eclosion from 10 μM, compared to the DMSO-treated group (Figures 2A, C). Notably, the duration of these processes remained unaffected (Figures 2B, D). Consequently, for subsequent experiments, we selected concentrations of 1, 10, or 100 μM as sublethal concentrations. Since eclosion rates fell below 50% at 10 and 100 µM (33.3% ± 12.6 at 10 μM and 40.0% ± 13.1 at 100 μM) (Supplementary Table S3), we exposed 4-day-old larvae to imidacloprid for 2 days. Subsequently, we monitored the daily activity of 5-day-old flies for 3 days using a DAM2 monitor. Despite the observed reductions in pupation and eclosion rates, our results showed no detectable impairment in locomotor activity in adults exposed to 1, 10 or 100 μM of imidacloprid for 2 days during the larval stage (Supplementary Figure S1A). Based on these findings, we selected these concentrations for further experiments, as they did not appear to affect fundamental behavioral abilities, particularly motor skills, in adult flies that successfully eclosed.

### 3.2 Larval and adult imidacloprid exposure affects olfactory learning and memory differently

**FIGURE 3:**
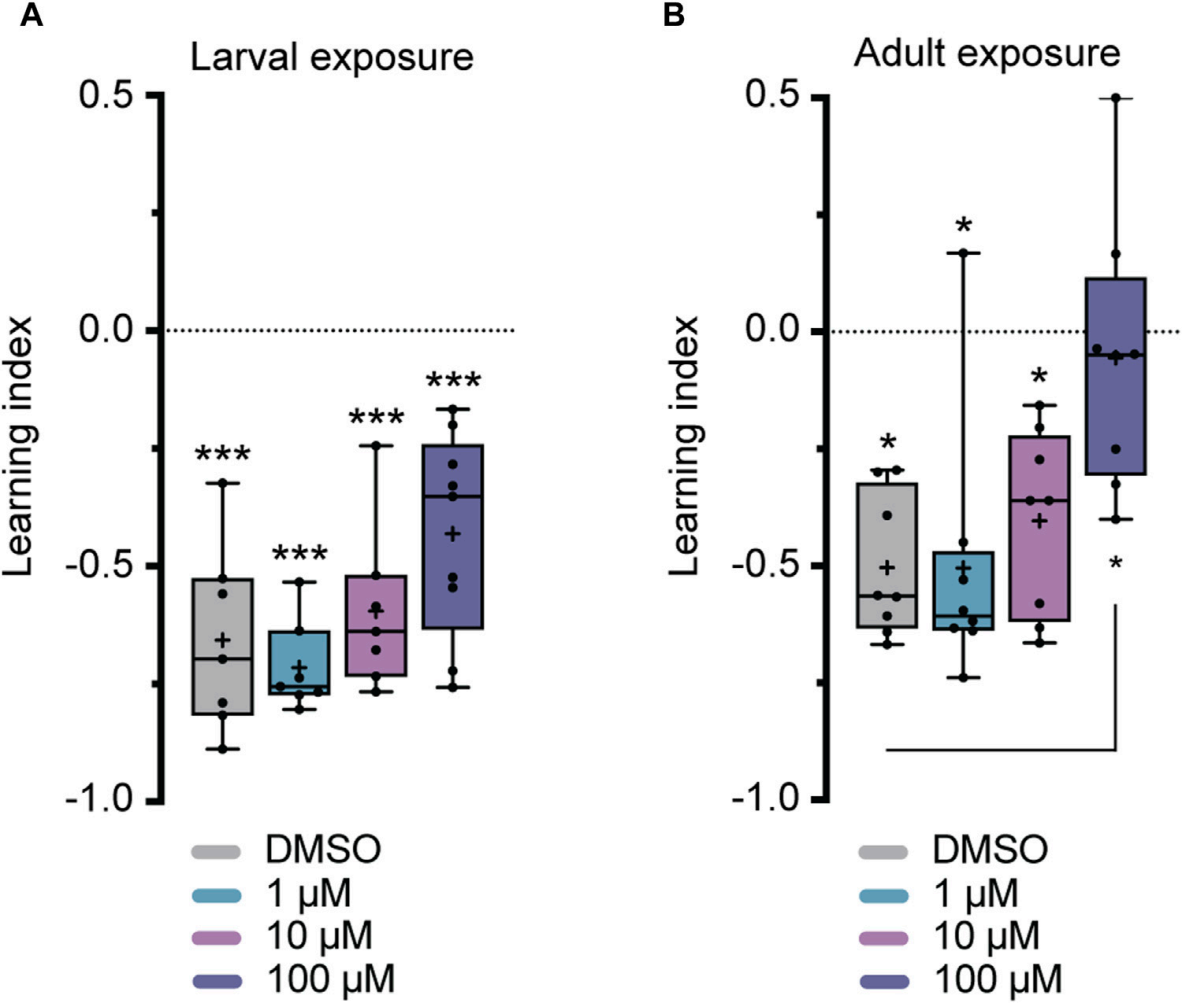
Effects of imidacloprid exposure on 3-min short-term memory in *Drosophila* larvae and adults. The 3-min memory was examined using a single-training paradigm (1x training) after the larvae or adults had been exposed to imidacloprid at concentrations of 0, 1, 10 or 100 μM. The memory was tested 3 min after training. **(A)** Larval exposure to imidacloprid did not significantly abolish 3-min memory. **(B)** Adult exposure to imidacloprid led to a significant abolishment in 3-min memory at 100 μM. Data are presented as Min-Max plots, with the boxes representing the interquartile range and the whiskers indicating 1.5 times the interquartile range. The median is depicted as a bold line, and the mean is represented by a cross within the box plot. Memory performance against the level of chance is indicated above the boxes with asterisks (*** for *p* < 0.001, ** for *p* < 0.01, * for *p* < 0.05). Statistical significance compared to the DMSO-treated group is indicated below the boxes (***p < 0.001, **p < 0.01, *p < 0.05). See Supplementary Tables S3, S5 for further statistical details.

**FIGURE 4:**
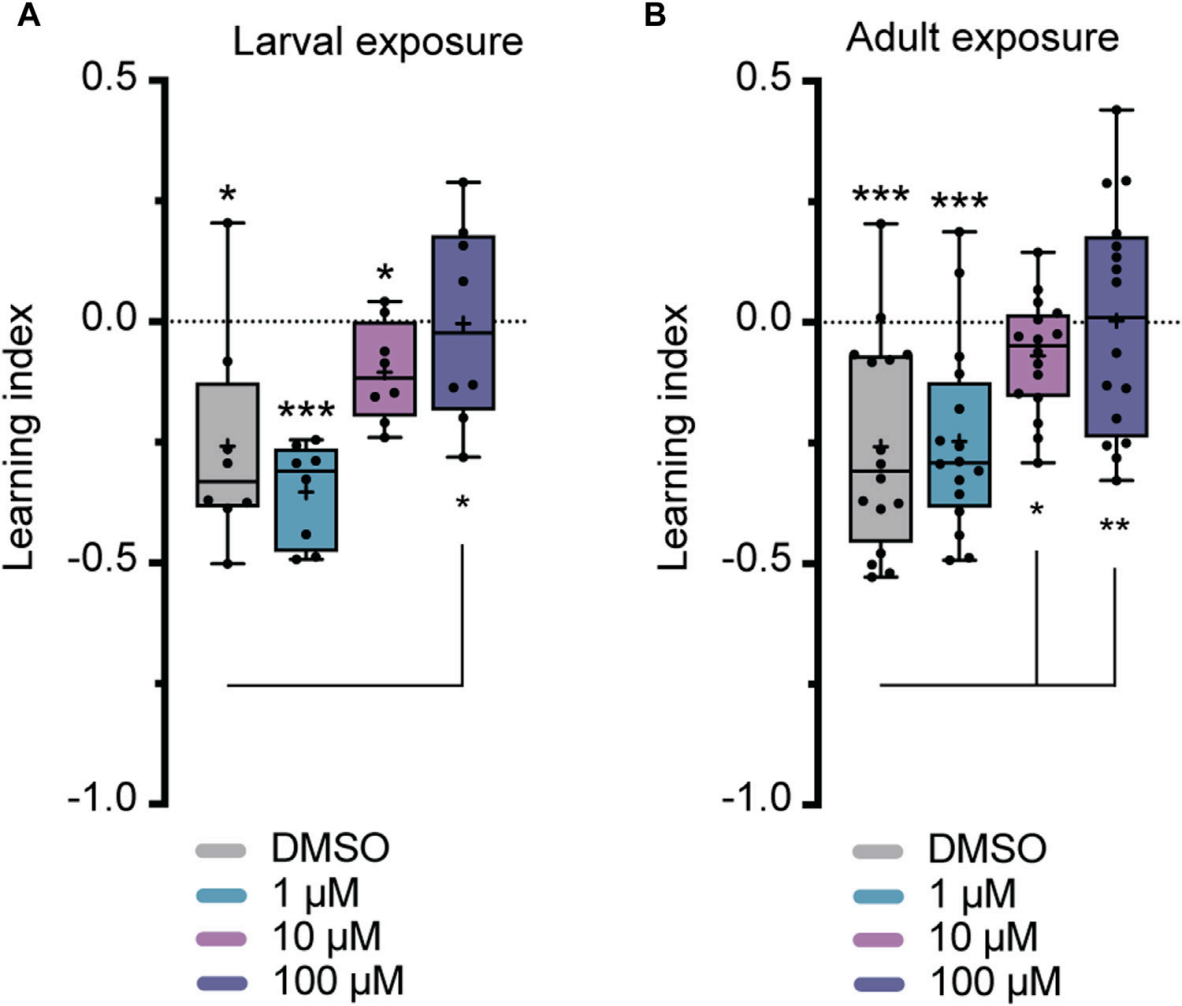
Effects of imidacloprid exposure on 24-h long-term memory in *Drosophila* larvae and adults. The 24-h memory was examined using a training paradigm with five trials at 15-min intervals (5x training) after the larvae or adults had been exposed to imidacloprid at concentrations of 0, 1, 10 or 100 μM. The memory was tested 24 h after training. **(A)** Larval exposure to imidacloprid showed a significant abolishment in 24-h memory at 100 μM. **(B)** Adult exposure to imidacloprid showed a significant abolishment of 24-h memory at 10 and 100 μM. Data are presented as Min-Max plots, with the boxes representing the interquartile range and the whiskers indicating 1.5 times the interquartile range. The median is depicted as a bold line, and the mean is represented by a cross within the box plot. Memory performance against the level of chance Is indicated above the boxes with asterisks (*** for *p* < 0.001, ** for *p* < 0.01, * for *p* < 0.05). Statistical significance compared to the DMSO-treated group is indicated below the boxes (***p < 0.001, **p < 0.01, *p < 0.05). See Supplementary Tables S3, S5 for further statistical details.

Flies were exposed to imidacloprid for a 2-day period during either the larval or adult stages before participating in olfactory shock-conditioning experiments, specifically targeting 3-min short-term memory (STM) and 24-h long-term memory (LTM) (Tully and Quinn, 1985). Larval exposure for 2 days did not significantly affect 3-min memory (Figure 3A), but abolished 24-h memory at the highest imidacloprid concentration (100 μM) (Figure 4A). Similarly, chronic exposure from the larval to the adult stage showed the same effect, leaving 3-min memory intact but abolished 24-h memory (Supplementary Figures S2B, S2C). However, when adults were exposed to the same high concentration (100 μM), it completely abolished their 3-min memory (Figure 3B) and interestingly a lower concentration of imidacloprid (10 μM) during adulthood was found to disrupt 24-h memory (Figure 4B) yet left 3-min memory unaffected (Figure 3B). In conclusion, exposure to imidacloprid affects both short-term and long-term memory in flies, with the impact varying by concentration and developmental stage of exposure. Notably, sensory responses to electric shock were unaffected across all concentrations tested, underscoring the specificity of imidacloprid-induced impairments in memory-related processes.

### 3.3 Imidacloprid exposure partially alters the dynamics of neurotransmitter release following olfactory stimulation

**FIGURE 5:**
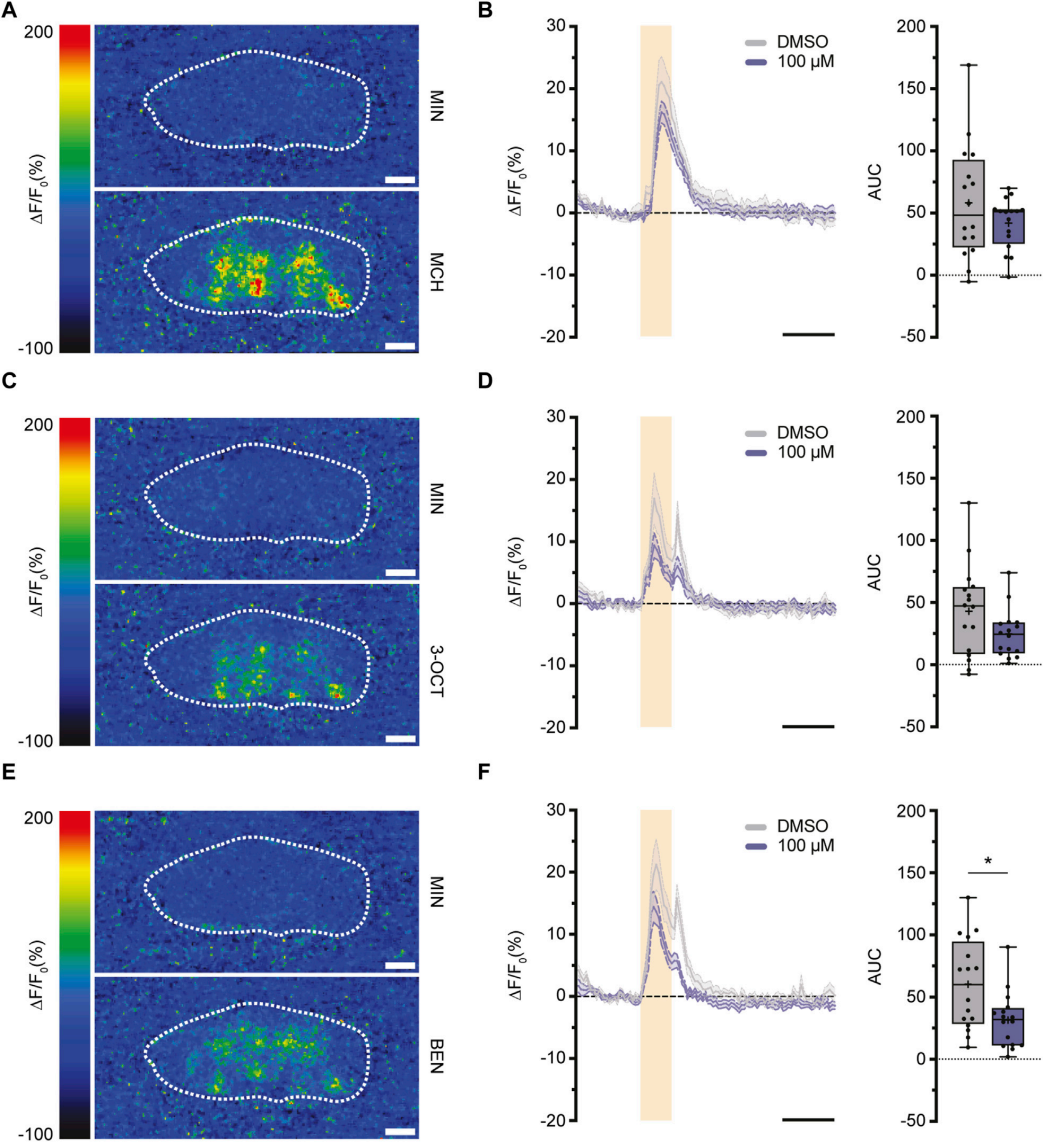
Exposure to imidacloprid in adult flies induces a partial reduction in acetylcholine dynamics in the MB calyx following odor stimulation. **(A,C,E)** Pseudocolor images representing the fluorescence response (ΔF/F₀) of GACh3.0 in a single fly, observed during exposure to either mineral oil (above) or the odorant MCH **(A)**, 3-OCT **(C)** or BEN **(E)** (below). White dashed circles mark the ROI of the calyx region used for quantification. **(B)** Exposure to 100 μM imidacloprid did not alter acetylcholine response after MCH stimulation. **(D)** Exposure to 100 μM imidacloprid did not alter acetylcholine response after 3-OCT stimulation. **(F)** Exposure to 100 μM imidacloprid reduced acetylcholine response after BEN stimulation. For **(B,D,F)** the odor-evoked activity traces are shown as the mean (solid line) and SEM (dashed line). Orange squares indicates a 3-s odor presentation, and the black line underneath indicates a 10-s unit of measurement. Normalized AUC is presented as Min-Max plots, with boxes representing the interquartile range and whiskers indicating 1.5 times the interquartile range. The median is depicted as a bold line, and the mean is represented by a cross within the box plot. Statistical significance between the DMSO-treated group and the imidacloprid-treated group is indicated above the boxes (***p < 0.001, **p < 0.01, *p < 0.05). See Supplementary Tables S3, S4 for further statistical details.

Having examined the varying impacts of imidacloprid exposure on memory formation in both larval and adult *Drosophila*, we shifted our focus to investigating the underlying neuronal mechanisms. The mushroom body (MB) is central to olfactory learning and memory processes, receiving sensory input through its calyx, where cholinergic synapses between PNs and KCs are located (Davis, 2023). Therefore, our study aimed next to find potential alterations in cholinergic transmitter activity between PNs and the MB calyx response to olfactory stimulation. Building on a prior finding of a significant reduction in the activity of individual olfactory neurons following imidacloprid exposure (Tatarko et al., 2023), we focused on assessing acetylcholine release during olfactory stimulation. We strategically opted for adult-specific imidacloprid exposure at the highest concentration (100 μM), given its pronounced impact on memory formation (Figures 3B, 4B). To investigate potential changes in acetylcholine release, we subjected 3-day-old adult flies to a 100 μM imidacloprid exposure for 2 days and initially assessed their behavioral responses to different odors. Utilizing three different odors—4-methylcyclohexanol (MCH), 3-octanol (3-OCT), and benzaldehyde (BEN)—we identified specific dilutions (1:10 for 3-OCT and MCH, and 1:100 for BEN) that induced significant odor responses post-imidacloprid exposure, albeit with a noticeable reduction compared to the DMSO control (Supplementary Figures S2A–S2C). Subsequently, we employed the GACh3.0 sensor, a modified G-protein-coupled receptor (GPCR) activation-based acetylcholine sensor (Jing et al., 2020), to indirectly measure acetylcholine release from the PNs onto the MB calyx. Using the MB-specific Gal4 driver line mb247-Gal4 (Supplementary Figure S2D), we expressed the GACh3.0 sensor throughout the entire MB, allowing comprehensive monitoring of acetylcholine dynamics in the MB calyx. Employing *in vivo* two-photon imaging, we examined acetylcholine release in response to 1:10 MCH, 1:10 OCT, and 1:100 BEN stimulation in the MB calyx (Figures 5A, C, E). Analysis of the data revealed no significant reduction in the activity of the GACh3.0 sensor upon odor stimulation with MCH and 3-OCT (Figures 5B, D). In contrast, a significant decrease in activity was observed for BEN (Figure 5F). In conclusion, our result showed that imidacloprid exposure induces dynamic changes in acetylcholine release between PNs and MB KCs. Importantly, however, our results showed differential effects of imidacloprid on acetylcholine release in response to different odors, emphasizing the need for further investigation into the intricate impact of imidacloprid on *Drosophila*’s olfactory circuitry.

### 3.4 Exposure to imidacloprid leads to a reduction in synaptic contacts between projection neurons and the mushroom body Kenyon cells

**FIGURE 6:**
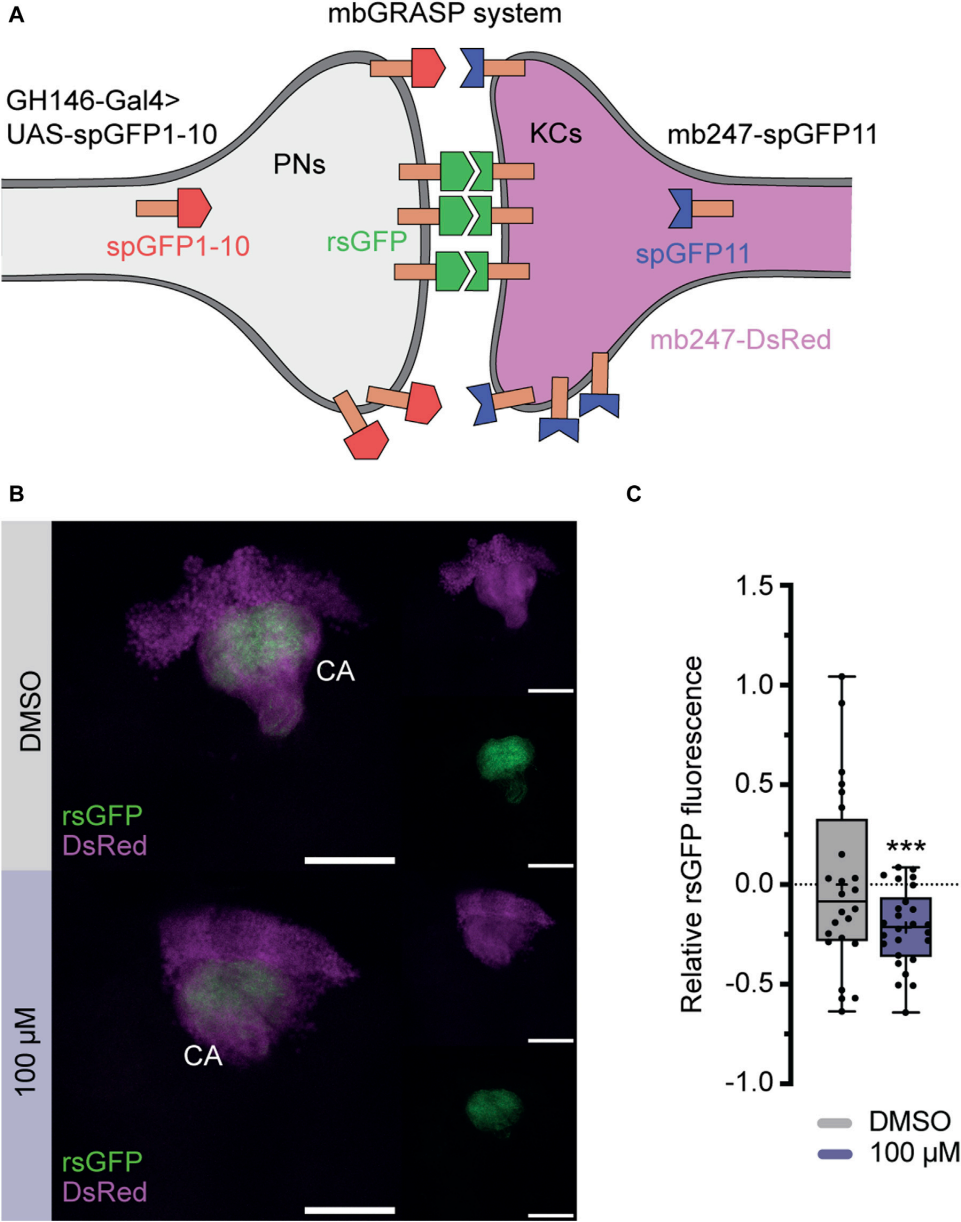
Exposure to imidacloprid in adult flies affects connectivity between PNs an MB KCs. **(A)** Schematic representation of the splitGFP reconstitution (rsGFP) technique to label sites of close proximity between PNs and KCs. KCs express mb247-splitGFP11 (depicted in blue), and the complementary GFP subunit (splitGFP1-10) is regulated by the PN-specific driver *GH146*-Gal4 (depicted in red). Additionally, all KCs are labeled with DsRed (depicted in pink). **(B)** Maximum projections of calyx regions from adult control (above) and imidacloprid-treated (below) flies show rsGFP fluorescence between olfactory PNs and KCs. **(C)** Exposure to imidacloprid (100 µM) at the adult stage resulted in a statistically significant reduction in rsGFP fluorescence in the MB calyx. Data are presented as Min-Max plots, with the boxes representing the interquartile range and the whiskers indicating 1.5 times the interquartile range. The median is depicted as a bold line, and the mean is represented by a cross within the box plot. Statistically significant deviations from baseline are indicated above the boxes with asterisks (***p < 0.001, **p < 0.01 and *p < 0.05). See Supplementary Table S3 for further statistical details. Calyx (Ca), Kenyon cells (KCs), mushroom body (MB), mbGRASP (mushroom body GRASP), projection neurons (PNs), rsGFP (reconstituted splitGFP).

To further investigate, whether imidacloprid disrupts not only the short-term response to acetylcholine for specific odors (Figure 5F), but also induces long-term changes in synaptic contacts within the PN-KC network, we employed the “green fluorescent protein reconstitution across synaptic partners” (GRASP) technique (Feinberg et al., 2008; Gordon and Scott, 2009). In this study, the technique detects changes in physical connections between KCs and PNs after exposure to imidacloprid. This involves quantitatively analyzing signal variations produced by complementary membrane-bound splitGFP proteins, originating from both the PNs and KCs. Specifically, one part of the splitGFP, co-expressed with the red fluorescent protein DsRed in KCs, was paired with second part expressed UAS-dependently via the PN-specific driver line *GH146*-Gal4 (Stocker et al., 1997; Pech et al., 2013) (Figure 6A). The resulting reconstituted splitGFP (rsGFP) served as a visual indicator of contacts between PNs and KCs, prominently visible in the calyx region (Figure 6B), allowing for a comparative assessment of differences in contacts within the PN-KC network after imidacloprid exposure. Focusing again on changes during the adult stage, 3-day-old animals were exposed to the highest concentration of imidacloprid (100 μM) for 2 days. The findings revealed a significant reduction in relative rsGFP fluorescence compared to the DMSO-only treated group (Figure 6C), signifying a notable decrease in contacts within the PN-KC network following exposure to imidacloprid. In conclusion, our study demonstrates that imidacloprid exposure leads to a substantial reduction in synaptic contacts between PNs and KCs in the MB calyx. This highlights the noteworthy impact of the pesticide on the neural circuitry associated with olfactory processing, as clearly evident in the PN-MB network.

## 4 Discussion

### 4.1 Impact of imidacloprid exposure on memory components in *Drosophila*

Unlike studies in honeybees, which have demonstrated differential effects of various neonicotinoids on memory components (Williamson and Wright, 2013), previous research in *Drosophila* has primarily shown a decrease in 1-h memory after exposure to neonicotinoids such as imidacloprid, clothianidin, and thiamethoxam (Tasman et al., 2021a). However, it is crucial to have a comprehensive understanding of the neurobiological effects of neonicotinoids on memory formation, given that memory formation is regulated by intricate molecular and cellular mechanisms (Widmann et al., 2018; Davis, 2023). Specifically, in the calyx, PNs facilitate the transmission of olfactory information across cholinergic synapses to intrinsic KCs (Zheng et al., 2022), which have recently been identified as undergoing circuit reorganization during memory consolidation (Baltruschat et al., 2021). Since neonicotinoids, including imidacloprid, interact with nicotinic acetylcholine receptors (nAChRs) (Matsuda et al., 2020), investigating the susceptibility of different memory components to neonicotinoids will enable a detailed analysis of cellular circuit vulnerabilities related to memory formation in response to these pesticides. Therefore, our study addressed this gap by investigating the nuanced effects of the neonicotinoid imidacloprid on different memory components in *Drosophila*. Importantly, our findings delineate a more severe impairment in 24-h memory relative to 3-min memory following a 2-day exposure to imidacloprid (Figures 3, 4). In instances of larval exposure, it is observed that 24-h memory is abolished only at the highest concentration tested, while the 3-min memory remains unaffected at any concentration. Conversely, during adult exposure, the onset of 24-h memory impairment is detected at a lower concentration of 10 μM, while 3-min memory shows susceptibility at 100 μM. This pattern not only highlights the heightened sensitivity of 24-h memory to imidacloprid but also details the intricate effects of concentration on memory processes. Our study’s scope, while limited, clearly indicates a pressing need for further research into how neonicotinoids influence memory. Future investigations should dissect the distinct effects on memory phases and the associated neural and molecular dynamics to deepen our understanding of their cognitive repercussions.

### 4.2 Developmental stage-dependent impact of imidacloprid exposure on olfactory memory in *Drosophila*

Given the dual role of nAChRs in both excitatory neurotransmission and neural development (Rosenthal and Yuan, 2021), it comes as no surprise that exposure to neonicotinoids during various developmental stages has already been shown to impact adult behavior and cognitive traits in other insects (Yang et al., 2012; Tan et al., 2015; Young et al., 2020). This pattern is similarly observed here, where olfactory memory is affected in both larval and adult stages of *Drosophila* (Figures 3, 4). However, a significant finding from this study is the notable difference in the effect of adult vs. larval exposure on memory formation. While we found that exposure to sublethal doses of imidacloprid during the late larval stage resulted in a reduction in pupation (Figure 2), it had only minimal effects on olfactory learning and memory formation in the surviving animals (Figures 3A, 4A). This holds true even if exposure to imidacloprid is extended from the larval until the adult stage (Supplementary Figures S1D, S1E). In contrast, exposure to imidacloprid specifically in adult flies provoked significant defects in olfactory learning and memory formation (Figures 3B, 4B). This differential response could be attributed to increased adaptive potential and structural flexibility during development, enabling coping with altered acetylcholine signaling. Given the genetic accessibility of *Drosophila*, its sequenced genome, and its demonstrated ability to develop resistance to insecticides (Adams et al., 2000; Hales et al., 2015; Homem et al., 2020; Matsuda et al., 2020), our findings provide a valuable avenue for investigating the underlying mechanisms responsible for increasing resistance to neonicotinoids at the molecular or genetic level. Such inquiries could offer valuable insight into how *Drosophila* might adapt to imidacloprid exposure, and the potential factors influencing resistance to this insecticide.

### 4.3 Impact of imidacloprid on the olfactory memory network in *Drosophila*

A critical region for olfactory learning and memory in *Drosophila* is the MB and its associated upstream neuronal circuits (Grabe and Sachse, 2018; Davis, 2023). Within this framework, intricate structures called microglomeruli (MGs) form complex synaptic structures between PNs and KCs (Yasuyama et al., 2002; Leiss et al., 2009). These MGs undergo a crucial transformation of olfactory information, shifting from a broadly tuned representation to a sparse one (Perez-Orive et al., 2002; Bhandawat et al., 2007; Turner et al., 2008; Honegger et al., 2011). This transformation is essential for reducing overlap, improving odor discrimination, and contributing to the complex process of olfactory learning (Olshausen and Field, 2004; Lin et al., 2014). In this context, neonicotinoids have been implicated in disrupting finely-tuned odor coding by affecting neurons within both central and peripheral olfactory pathways (Palmer et al., 2013; Andrione et al., 2016; Tatarko et al., 2023). Consistent with this, we have additionally demonstrated a significant reduction in cholinergic activity within the PN-MB network using a novel high-speed acetylcholine sensor (Jing et al., 2020), particularly with the odor BEN (Figure 5F). However, this change is not consistent for all odors presented, likely due to odor-specific variability in the activity of PNs and anterior paired lateral (APL) neurons at the calyx (Prisco et al., 2021). Therefore, additional experiments, such as testing more odor concentrations, are warranted to provide a comprehensive understanding of the changes in cholinergic activity. Moreover, we have gained preliminary evidence suggesting that exposure to imidacloprid during adulthood not only modifies cholinergic signal activity but also diminishes synaptic contacts between the MB and PNs (Figure 6C). However, it is important to note that these findings are preliminary, and further studies are required to determine whether exposure to imidacloprid indeed induces long-term changes at the synapses. Nevertheless, it should be emphasised that our assessment utilized only one concentration of imidacloprid and involved only a 2-day exposure period. For future studies, an intriguing avenue could involve exploring changes in neuronal connectivity and acetylcholine signaling in response to different odors, various sublethal imidacloprid concentrations, and diverse exposure durations. With this, our study can be seen as a starting point in understanding how neonicotinoids impact PN-MB network and neurotransmitter dynamics in *Drosophila*.

### 4.4 Summary

Our study sheds light on the diverse impacts of imidacloprid exposure on memory components in *Drosophila*, emphasizing the need for detailed exploration of the specific neuronal pathways involved in memory impairment following neonicotinoid exposure. We observed significant differences in memory formation between larval and adult stages, suggesting heightened adaptability during developmental stages. Furthermore, imidacloprid exposure resulted in variations in odor responses, changes in cholinergic activity, and a decrease in synaptic contacts between PNs and KCs. While our study offers valuable insights, it is essential to acknowledge its limitations in assessing neuronal connectivity and acetylcholine signaling. Therefore, further investigations exploring these aspects in response to different odors, varied sublethal concentrations, and diverse exposure durations are warranted. Overall, our findings provide a framework for a deeper understanding of neonicotinoid impacts on memory formation and olfactory circuits in *Drosophila*, offering valuable insights for future research.

## Data Availability

The raw data supporting the conclusion of this article will be made available by the authors, without undue reservation.

## Supplementary material

SUPPLEMENTARY FIGURE S1
**(A)** Influence of laval stage imidacloprid exposure on adult fly activity. Locomotion was monitored over 3 consecutive days in 3-5 days old male flies exposed to imidacloprid during their larval stage, under a 12-hr light/dark cycle. Activity events were counted over a 24-hr period. Cumulative values for each fly over 3 days were averaged to calculate a final mean per group. No significant impact on adult fly activity was observed following larval stage imidacloprid exposure. Data are presented as bar graphs with mean values and SEM represented by error bars. Statistical significance is indicated above the graphs (*** for p < 0.001, ** for p < 0.01, * for p < 0.05). See Supplementary Tables S3, S5 for further statistical details. **(B,C)** Effects of chronic imidacloprid exposure on 3-minute and 24-hour memory in Drosophila larvae and adults. The treatment regimen started on day 4 of larval development and persisted into adulthood. While no significant impairment in 3-minute memory was observed **(B)**, a significant abolishment in 24-hour memory was evident at 10 and 100 μM **(C)**. **(D,E)** Flies exposed to imidacloprid either during the larval stage **(D)** or adult stage **(E)** did not exhibit an abolishment in perceiving the electric shocks. Data are presented as Min-Max plots, with the boxes representing the interquartile range and the whiskers indicating 1.5 times the interquartile range. The median is depicted as a bold line, and the mean was represented by a cross within the box plot. Perception of electric shocks and memory performance against the level of chance is indicated above the boxes with asterisks (*** for p < 0.001, ** for p < 0.01, * for p < 0.05). Statistical significance compared to the DMSO-treated group is indicated below the boxes. See Supplementary Tables S3, S5 for further statistical details.

SUPPLEMENTARY FIGURE S2 Olfactory perception of 3-OCT, MCH, and BEN following adult exposure to 100 μM imidacloprid. Olfactory perception was completely abolished in animals at a 1:100 and 1:1000 dilution of 3-OCT. **(B)** Olfactory perception was completely abolished in animals at a 1:100 and 1:1000 dilution of MCH. **(C)** Olfactory perception was completely abolished in animals at a 1:1000 dilution of BEN. **(D)** A comprehensive view of all MB lobes was obtained through a maximum z-projection using confocal microscopy, induced by the mb247-Gal4 driver line. This driver line regulates the expression pattern of membrane-bound mCD8-GFP and the presynaptic marker nSyb-GFP. Gal4-expressing cells were stained with anti-GFP (left), while anti-DLG was used to stain the neuropils (middle). The right panel presents an overlay of both images. Scale bars: 50 µm. Data are presented as Min-Max plots, with the boxes representing the interquartile range and the whiskers indicating 1.5 times the interquartile range. The median is depicted as a bold line, and the mean is represented by a cross within the box plot. Olfactory perception against the level of chance is indicated above the boxes with asterisks (*** for p < 0.001, ** for p < 0.01, * for p < 0.05). Statistical significance between the DMSO-treated group and the imidacloprid-treated group is indicated above the boxes (*** p < 0.001, ** p < 0.01, * p < 0.05). See Supplementary Tables S3, S4 for further statistical details.
