# Supplementary figures and images for: Exploring neonicotinoid effects on Drosophila: insights into olfactory memory, neurotransmission, and synaptic connectivity
Source: Front Physiol. 2024 Mar 14;15:1363943. doi: 10.3389/fphys.2024.1363943 (PMC10973132; doi:10.3389/fphys.2024.1363943)

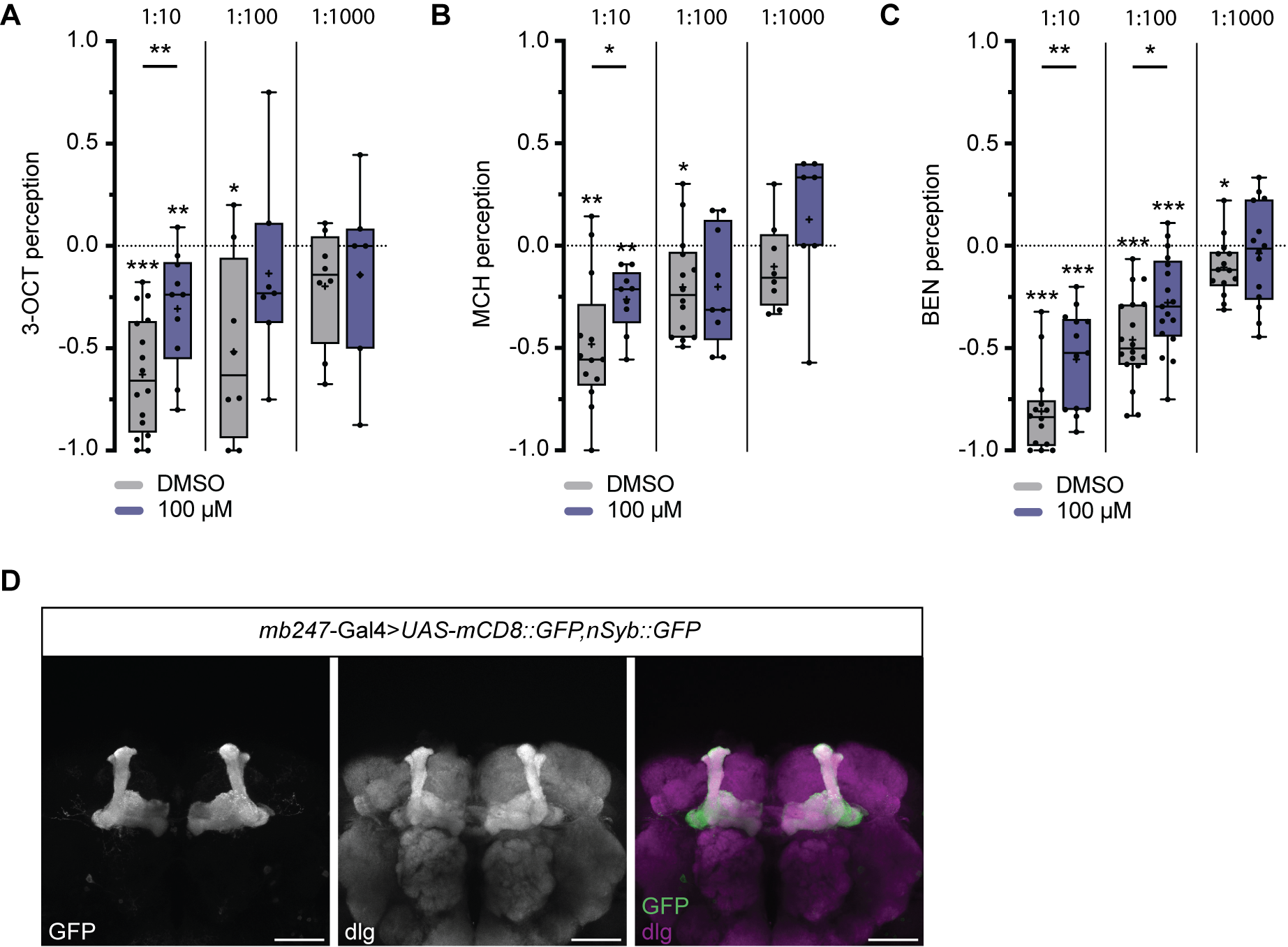

Supplement: Supplementary file 1 [file Image2.TIF]

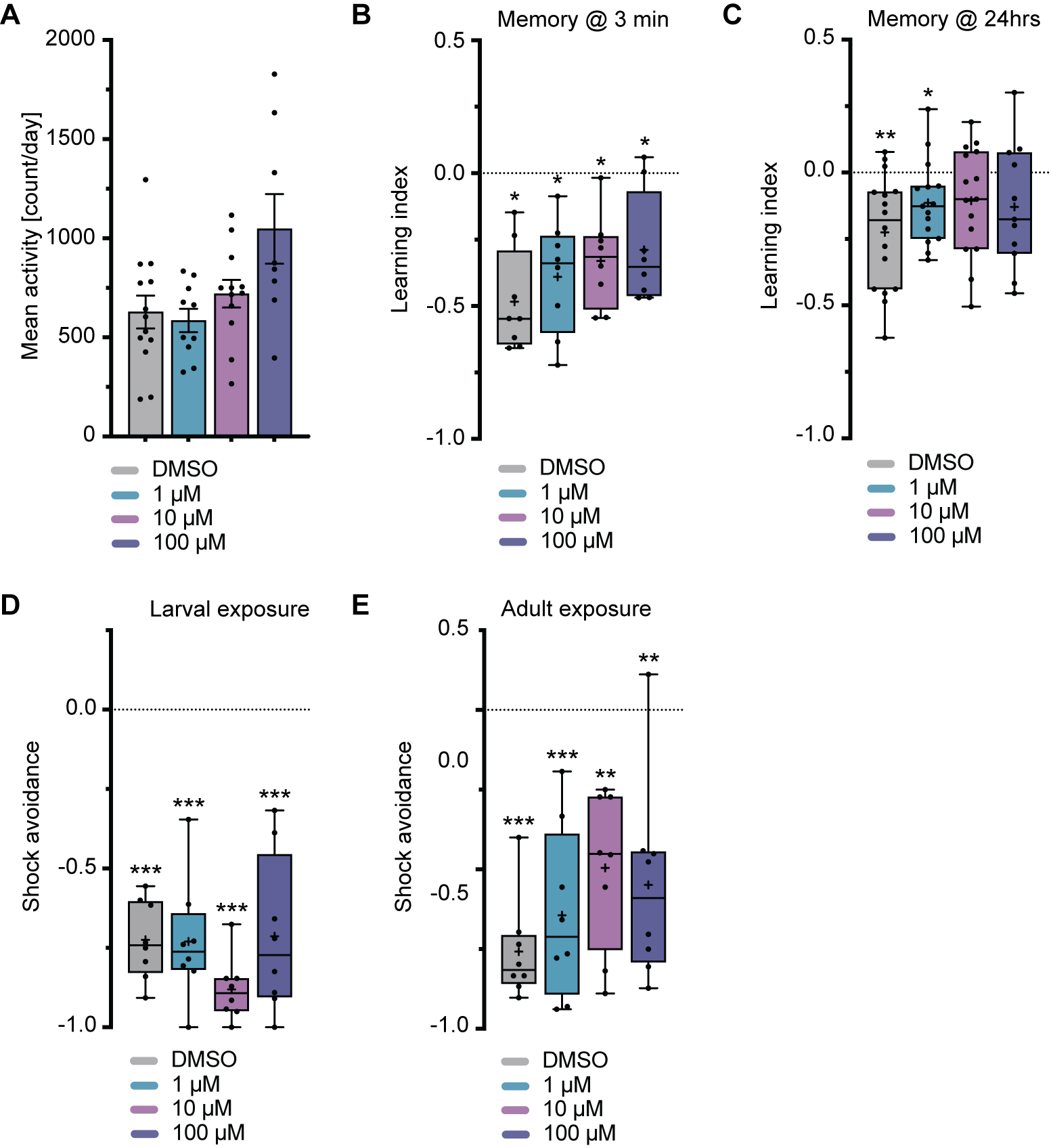

Supplement: Supplementary file 2 [file Image1.TIF]
