# Supplementary material for: Exploring neonicotinoid effects on Drosophila: insights into olfactory memory, neurotransmission, and synaptic connectivity
Source: Front Physiol. 2024 Mar 14;15:1363943. doi: 10.3389/fphys.2024.1363943 (PMC10973132; doi:10.3389/fphys.2024.1363943)
Supplement: Supplementary file 3 [file DataSheet1.docx]

Supplementary Material

### Supplementary Table 1: Key resource table.

| Reagent or resource | Source | Identifier |
| --- | --- | --- |
| Chemicals, peptides, and recombinant proteins | | |
| 3-octanol | Sigma-Aldrich | Cat#218405; CAS: 589-98-0 |
| 4-methylcyclohexanol | Sigma-Aldrich | Cat#153095; CAS: 589-91-3 |
|  |  |  |
| Agarose | Sigma-Aldrich | Cat#A9539; CAS: 9012-36-6 |
| Benzaldehyde | Sigma-Aldrich | Cat#B1334; CAS: 100-52-7 |
| Bovine Serum Albumin (BSA) Fraction V | Carl Roth GmbH+Co.KG | Cat#8076; CAS: 9048-46-8 |
| Apple juice (clear, 100 % fruit content) | tegut... gute Lebensmittel GmbH & Co. KG | #4305720071818 |
| L-ascorbic acid | Sigma-Aldrich | Cat#A7506; CAS: 50-81-7 |
| Brewer’s yeast | Gewürzmühle Brecht | #03462-0500 |
| Dimethyl sulphoxide (DMSO) | Sigma-Aldrich | Cat#D2650; CAS: 67-68-5 |
| Imidacloprid | Sigma-Aldrich | Cat#37894; CAS: 138261-41-3 |
| Indigo carmine | Sigma-Aldrich | Cat#860-22-0; CAS: 860-22-0 |
| Mineral oil | Sigma-Aldrich | Cat#M8410; CAS: 8042-47-5 |
| Normal Goat Serum (NGS) | Invitrogen | Cat#31873; RRID: AB_2532167 |
| Paraformaldehyde | Carl Roth GmbH+Co.KG | Cat#0335; CAS: 30525-89-4 |
| Colour fix (strawberry coloured) | Ruth GmbH & Co.KG | Cat#9805 |
| Sucrose | Millipore | Cat#84100; CAS: 57-50-1 |
| Triton® X 100 | Carl Roth GmbH+Co.KG | Cat#3501; CAS: 9002-93-1 |
| **Critical commercial assays** | | |
| VECTASHIELD Mounting Medium | Vector laboratories | Cat#H-1000 |
| **Software and algorithms** | | |
| Adobe Illustrator 2022 | Adobe | <https://www.adobe.com/products/illustrator.html>; RRID: SCR_010279 |
| DAMSystem311 | TriKinetics Inc. | <https://trikinetics.com/> |
| Fiji | Schindelin et al. 2012 | [http://fiji.sc](http://fiji.sc); RRID: SCR_002285 |
| GraphPad Prism | GraphPad Software | <http://www.graphpad.com/>; RRID:SCR_002798 |
| ImageJ | National Institutes of Health | <https://imagej.net/ij/>; RRID:SCR_003070 |
| Leica Application Suite X (LAS X) | Leica Microsystem | [https://www.leica-microsystems.com/products/microscope-software/details/product/leica-las-x-ls/](https://www.leica-microsystems.com/products/microscope-software/details/product/leica-las-x-ls/); RRID: SCR_013673 |
| OriginPro 2020 | Origin Lab | [http://www.originlab.com/index.aspx?go=PRODUCTS/Origin](http://www.originlab.com/index.aspx?go=PRODUCTS/Origin); RRID:SCR_014212 |

### Supplementary Table 2: *Drosophila* strains used in this study.

| Genotype^1^ | Source | Reference |
| --- | --- | --- |
| Wilde-type *Canton-S* | A gift from A. Fiala |  |
| *w^1118^* | A gift from A. Fiala |  |
| *mb247-DsRed;mb247-splitGFP11, UAS-splitGFP1-10* (II,III) | A gift from A. Fiala | Pech et al., 2013 |
| *y^1^, w^1118^;P{w^+mW.hs^=GawB}GH146* (II) | BDSC 30026 | Stocker et al., 1997 |
| *w^1118^;;mb247:mCherry-CAAX* (III) | A gift from O. Kobler | Kobler et al., 2020 |
| *UAS-GACh3.0* (II) | BDSC#86549 | Jing et al., 2020 |
| *mb247-Gal4*/*CyO* (II) | A gift from E.J. Hong |  |

^1^Includes all genotypes used in this study, including those that do not appear in figures.

**Supplementary Table 3: Sample size, mean±s.e.m. and statistical details of test against chance level.**

|  | Group^1^ | n | Mean±s.e.m | Shapiro-Wilk test^2^ | Statistical description^3^ | Adjusted p-value^4^ |
| --- | --- | --- | --- | --- | --- | --- |
| Figure 2A | 1 | 15 | 100±0.00 |  |  |  |
|  | 2 | 10 | 80.0±13.3 | W=0.509, p<0.001 |  |  |
|  | 3 | 13 | 84.6±10.4 | W=0.446, p<0.001 |  |  |
|  | 4 | 15 | 80.0±10.7 | W=0.499, p<0.001 |  |  |
|  | 5 | 15 | 86.7±09.09 | W=0.413, p<0.001 |  |  |
|  | 6 | 15 | 66.7±12.6 | W=0.603, p<0.001 |  |  |
|  | 7 | 15 | 46.7±13.3 | W=0.643, p<0.001 |  |  |
|  | 8 | 13 | 7.69±07.69 | W=0.311, p<0.001 |  |  |
| Figure 2B | 1 | 15 | 182±4.57 | W=0.755, p=0.001 |  |  |
|  | 2 | 8 | 192±9.07 | W=0.860, p=0.120 |  |  |
|  | 3 | 10 | 209±6.25 | W=0.841, p=0.045 |  |  |
|  | 4 | 11 | 194±5.07 | W=0.822, p=0.018 |  |  |
|  | 5 | 11 | 188±4.36 | W=0.774, p=0.004 |  |  |
|  | 6 | 10 | 185±6.25 | W=0.781, p=0.008 |  |  |
|  | 7 | 7 | 192±10.5 | W=0.856, p=0.139 |  |  |
|  | 8 | 1 | 192±0.00 | n too small |  |  |
| Figure 2C | 1 | 15 | 0.93.3±6.67 | W=0.284, p<0.001 |  |  |
|  | 2 | 10 | 0.80.0±13.3 | W=0.509, p<0.001 |  |  |
|  | 3 | 13 | 0.73.3±11.8 | W=0.561, p<0.001 |  |  |
|  | 4 | 15 | 0.66.7±12.6 | W=0.603, p<0.001 |  |  |
|  | 5 | 15 | 0.66.7±12.6 | W=0.603, p<0.001 |  |  |
|  | 6 | 15 | 0.33.3±12.6 | W=0.603, p<0.001 |  |  |
|  | 7 | 15 | 0.40.0±13.1 | W=0.630, p<0.001 |  |  |
|  | 8 | 13 | 0.07.69±7.69 | W=0.311, p<0.001 |  |  |
|  | **Group^1^** | **n** | **Mean±s.e.m** | **Shapiro-Wilk test^2^** | **Statistical description^3^** | **Adjusted p-value^4^** |
| Figure 2D | 1 | 14 | 278±4.14 | W=0.688, p<0.001 |  |  |
|  | 2 | 7 | 288±0.00 |  |  |  |
|  | 3 | 7 | 288±0.00 |  |  |  |
|  | 4 | 11 | 284±2.93 | W=0.486, p<0.001 |  |  |
|  | 5 | 8 | 273±7.77 | W=0.693, p=0.002 |  |  |
|  | 6 | 4 | 276±6.93 | W=0.729, p=0.024 |  |  |
|  | 7 | 5 | 278±5.88 | W=0.684, p=0.006 |  |  |
|  | 8 | 1 | 264±0.00 |  |  |  |
| Figure 3A | 1 | 7 | -0.657±0.075 | W=0.946, p=0.6896 | t=8.7, df=6, p<0.001 | <0.001, *** |
|  | 2 | 7 | -0.715±0.036 | W=0.831, p=0.0819 | t=19.7, df=6, p<0.001 | <0.001, *** |
|  | 3 | 7 | -0.595±0.067 | W=0.874, p=0.1999 | t=8.95, df=6, p<0.001 | <0.001, *** |
|  | 4 | 9 | -0.431±0.072 | W=0.921, p=0.4000 | t=5.98, df=8, p<0.001 | <0.001, *** |
| Figure 3B | 1 | 8 | -0.504±0.0538 | W=0.855, p=0.106 | W=-36.0, p=0.008 | 0.031, * |
|  | 2 | 8 | -0.504±0.101 | W=0.696, p=0.002 | W=-34.0, p=0.016 | 0.031, * |
|  | 3 | 8 | -0.404±0.0698 | W=0.903, p=0.305 | W=-36.0, p=0.008 | 0.031, * |
|  | 4 | 8 | -0.0552±0.102 | W=0.926, p=0.483 | W=-12.0, p=0.461 | 0.461, * |
| Figure 4A | 1 | 8 | -0.259±0.0788 | W=0.860, p=0.121 | t=3.28, df=7, p=0.013 | 0.040, * |
|  | 2 | 8 | -0.354±0.0365 | W=0.845, p=0.086 | t=9.69, df=7, p<0.001 | <0.001, *** |
|  | 3 | 8 | -0.105±0.0360 | W=0.949, p=0.704 | t=2.91, df=7, p=0.023 | 0.045, * |
|  | 4 | 8 | -0.0041s±0.0736 | W=0.924, p=0.466 | t=0.0563, df=7, p=0.957 | 0.957, ns |
| Figure 4B | 1 | 16 | -0.257±0.0548 | W=0.923, p=0.188 | t=4.70, df=15, p<0.001 | <0.001, *** |
|  | 2 | 16 | -0.247±0.0487 | W=0.920, p=0.169 | t=5.08, df=15, p<0.001 | <0.001, *** |
|  | 3 | 16 | -0.0691±0.0295 | W=0.985, p=0.991 | t=2.34, df=15, p=0.033 | 0.066, ns |
|  | 4 | 16 | 0.00309±0.05958 | W=0.939, p=0.335 | t=0.0518, df=15, p=0.959 | 0.960, ns |
| Figure 5C | 1 | 24 | 0.00±0.0915 | W=0.933, p=0.115 | t=0.00, df=23, p>0.999 | >0.999, ns |
|  | 2 | 26 | -0.216±0.0383 | W=0.969, p=0.595 | t=5.64, df=25, p<0.001 | <0.001, *** |
| Figure 6B | 1 | 16 | 58.2±11.5 | W=0.946, p=0.427 |  |  |
|  | 2 | 16 | 42.0±5.11 | W=0.920, p=0.165 |  |  |
| Figure 6D | 1 | 16 | 43.0±9.25 | W=0.946, p=0.433 |  |  |
|  | 2 | 16 | 24.7±4.84 | W=0.899, p=0.076 |  |  |
| Figure 6F | 1 | 16 | 60.3±9.10 | W=0.939, p=0.337 |  |  |
|  | **Group^1^** | **n** | **Mean±s.e.m** | **Shapiro-Wilk test^2^** | **Statistical description^3^** | **Adjusted p-value^4^** |
|  | 2 | 16 | 31.5±5.62 | W=0.913, p=0.132 |  |  |
| Figure | 1 | 13 | 628.3±83.01 | W=0.951, p=0.617 |  |  |
| S1A | 2 | 10 | 585.4±59.14 | W=0.924, p=0.394 |  |  |
|  | 3 | 12 | 720.4±69.66 | W=0.951, p=0.658 |  |  |
|  | 4 | 8 | 1048±175.6 | W=0.924, p=0.459 |  |  |
| Figure | 1 | 8 | -0.484±0.0682 | W=0.840, p=0.075 | W=-36.0, p=0.008 | 0.031, * |
| S1B | 2 | 8 | -0.390±0.0756 | W=0.963, p=0.835 | W=-36.0, p=0.008 | 0.031, * |
|  | 3 | 8 | -0.331±0.0619 | W=0.942, p=0.626 | W=-36.0, p=0.008 | 0.031, * |
|  | 4 | 8 | -0.288±0.0738 | W=0.815, p=0.042 | W=-30.0, p=0.039 | 0.039, * |
| Figure | 1 | 16 | -0.232±0.0516 | W=0.940, p=0.347 | t=4.50, df=15, p<0.001 | 0.0016, ** |
| S1C | 2 | 15 | -0.114±0.0403 | W=0.955, p=0.604 | t=2.83, df=14, p=0.013 | 0.0397, * |
|  | 3 | 15 | -0.0499±0.0513 | W=0.919, p=0.185 | t=0.973, df=14, p=0.347 | 0.347, ns |
|  | 4 | 11 | -0.129±0.0704 | W=0.966, p=0.840 | t=1.84, df=10, p=0.096 | 0.183, ns |
| Figure | 1 | 8 | -0.724±0.0439 | W=0.952, p=0.727 | t=16.5, df=7, p<0.001 | <0.001, *** |
| S1D | 2 | 8 | -0.730±0.0670 | W=0.909, p=0.347 | t=10.9, df=7, p<0.001 | <0.001, *** |
|  | 3 | 8 | -0.881±0.0350 | W=0.900, p=0.291 | t=25.2, df=7, p<0.001 | <0.001, *** |
|  | 4 | 8 | -0.881±0.0877 | W=0.907, p=0.331 | t=8.14, df=7, p<0.001 | <0.001, *** |
| Figure | 1 | 8 | -0.758±0.0564 | W=0.798, p=0.027 | W=-36.0, p=0.008 | 0.031, * |
| S1E | 2 | 8 | -0.644±0.0953 | W=0.921, p=0.440 | W=-36.0, p=0.008 | 0.031, * |
|  | 3 | 8 | -0.495±0.0872 | W=0.871, p=0.153 | W=-36.0, p=0.008 | 0.031, * |
|  | 4 | 8 | -0.549±0.112 | W=0.863, p=0.127 | W=-34.0, p=0.016 | 0.031, * |
| Figure  S2A | 1 | 16 | -0.628±0.0716 | W=0.924, p=0.196 | t=8.761, df=15, p<0.001 | <0.001, *** |
| 1:10 | 2 | 10 | -0.307±0.0905 | W=0.948, p=0.643 | t=3.393, df=9, p=0.008 | p=0.0078, ** |
| 1:100 | 1 | 8 | -0.517±0.159 | W=0.907, p=0.332 | t=3.24, df=7, p=0.0142 | 0.0282, * |
|  | 2 | 7 | -0.135±0.176 | W=0.908, p=0.382 | t=0.765, df=6, p=0.473 | 0.473, ns |
| 1:1000 | 1 | 8 | -0.197±0.101 | W=0.877, p=0.177 | t=1.95, df=7, p=0.092 | 0.165, ns |
|  | 2 | 7 | -0.141±0.162 | W=0.944, p=0.672 | t=0.874, df=6, p=0.416 | 0.438, ns |
| Figure | 1 | 13 | -0.482±0.0906 | W=0.924, p=0.284 | W=-83.0, p=0.0017 | 0.0034, ** |
| S2B  1:10 | 2 | 9 | -0.262±0.0519 | W=0.917, p=0.370 | W=-45.0, p=0.0039 | 0.0029, ** |
|  | **Group^1^** | **n** | **Mean±s.e.m** | **Shapiro-Wilk test^2^** | **Statistical description^3^** | **Adjusted p-value^4^** |
| 1:100 | 1 | 14 | -0.203±0.0671 | W=0.920, p=0.223 | t=3.02, df=10, p=0.010 | 0.0197, * |
|  | 2 | 9 | -0.201±0.0951 | W=0.888, p=0.191 | t=2.12, df=8, p=0.067 | 0.067, ns |
| 1:1000 | 1 | 8 | -0.102±0.07587 | W=0.927, p=0.488 | W=-20.0, p=0.195 | 0.353, ns |
|  | 2 | 7 | 0.128±0.1340 | W=0.793, p=0.0352 | W=5.00, p=0.688 | 0.688, ns |
| Figure | 1 | 14 | -0.810±0.0549 | W=0.830, p=0.012 | W=-105.0, p<0.001 | <0.001, *** |
| S2C  1:10 | 2 | 13 | -0.555±0.0672 | W=0.900, p=0.135 | W=-91.00, p<0.001 | <0.001, *** |
| 1:100 | 1 | 18 | -0.459±0.0516 | W=0.962, p=0.643 | t=8.90, df=17, p<0.001 | <0.001, *** |
|  | 2 | 17 | -0.278±0.0573 | W=0.982, p=0.972 | t=4.85, df=16, p<0.001 | <0.001, *** |
| 1:1000 | 1 | 14 | -0.106±0.0378 | W=0.960, p=0.729 | t=2.79, df=13, p=0.015 | 0.0304, * |
|  | 2 | 14 | -0.0371±0.0666 | W=0.954, p=0.620 | t=0.557, df=13, p=0.587 | 0.587, ns |

^1^Numbers correspond to boxplots from left to right. ^2^Significance level was set to 𝛼=0.05, p≥0.05 indicates normal distribution. ^3^Two-tailed one-sample t-test (^a^) or Wilcoxon signed-rank test (^b^). Theoretical mean was µ=0.000. ^4^Holm-Sidak adjusted p-values. Significance level was set to 𝛼=0.05; ns p≥0.05, * p<0.05, ** p<0.01, *** p<0.001.

### Supplementary Table 4: Statistical details of unpaired t-test or Mann-Whitney test.

|  | Group^1^ | Statistical test | Statistical description | p-value^3^ |
| --- | --- | --- | --- | --- |
| Figure 5B |  | Mann-Whitney test^2^ | U=104 | 0.381, ns |
| Figure 5D |  | Mann-Whitney test^2^ | U=93 | 0.196, ns |
| Figure 5F |  | Unpaired t-test^2^ | t=2.68, df=30 | 0.012, * |
| Figure S2A  1:10 |  | Mann-Whitney test^2^ | U=30 | 0.0070, ** |
| Figure S2A  1:100 |  | Mann-Whitney test^2^ | U=15.5 | 0.1632, ns |
| Figure S2A  1:1000 |  | Mann-Whitney test^2^ | U=23 | 0.5913, ns |
| Figure S2B  1:10 |  | Mann-Whitney test^2^ | U=29 | 0.0497, * |
| Figure S2B  1:100 |  | Mann-Whitney test^2^ | U=59.5 | 0.8410, ns |
| Figure S2B  1:1000 |  | Mann-Whitney test^2^ | U=12 | 0.0674, ns |
| Figure S2C  1:10 |  | Mann-Whitney test^2^ | U=37 | 0.0077, ** |
| Figure S2C  1:100 |  | Mann-Whitney test^2^ | U=87 | 0.0293, * |
|  | **Group^1^** | **Statistical test** | **Statistical description** | **p-value^3^** |
| Figure S2D  1:1000 |  | Mann-Whitney test^2^ | U=77 | 0.3457, ns |

^1^Numbers correspond to boxplots from left to right. ^2^Two-tailed. ^3^Significance level was set to 𝛼=0.05; ns p≥0.05, * p<0.05, ** p<0.01, *** p<0.001

### Supplementary table 5: Statistical details of ordinary one-way ANOVA and non-parametric Kruskal-Wallis test.

|  | Statistical description^1^ | Significant^2^ | Multiple comparison tests^3^ | Bartlett’s test^4^ |  |
| --- | --- | --- | --- | --- | --- |
| Figure 2A | | H=38.1, p<0.001^b^ | *** | 1 vs 2, p>0.9999, ns |  |
|  | |  |  | 1 vs 3, p>0.9999, ns |  |
|  | |  |  | 1 vs 4, p>0.9999, ns |  |
|  | |  |  | 1 vs 5, p>0.9999, ns |  |
|  | |  |  | 1 vs 6, p=0.3407, ns |  |
|  | |  |  | 1 vs 7, p=0.0113, * |  |
|  | |  |  | 1 vs 8, p<0.001, *** |  |
| Figure 2B | | H=11.7, p=0.1103^b^ | ns |  |  |
| Figure 2C | | H=30.9, p<0.001^b^ | *** | 1 vs 2, p>0.9999, ns |  |
|  | |  |  | 1 vs 3, p>0.9999, ns |  |
|  | |  |  | 1 vs 4, p=0.9893, ns |  |
|  | |  |  | 1 vs 5, p=0.9893, ns |  |
|  | |  |  | 1 vs 6, p=0.0065, ** |  |
|  | |  |  | 1 vs 7, p=0.0228, * |  |
| Figure 2D | | H=10.5, p=0.1638^b^ | ns |  |  |
| Figure 3A | | H=8.549, p=0.0359^b^ | * | 1 vs 2, p>0.999, ns |  |
|  | |  |  | 1 vs 3, p>0.999, ns |  |
|  | |  |  | 1 vs 4, p=0.0896, ns |  |
| Figure 3B | | H=11.74, p=0.0083^b^ | * | 1 vs 2, p>0.999, ns |  |
|  | |  |  | 1 vs 3, p>0.999, ns |  |
|  | |  |  | 1 vs 4, p=0.0142, * |  |
| Figure 4A | | F(3,28)=6.82, p=0.0014^a^ | ** | 1 vs 2, p=0.5435, ns | p=0.0805 |
|  | |  |  | 1 vs 3, p=0.1881, ns |  |
|  | |  |  | 1 vs 4, p=0.0147, * |  |
| Figure 4B | | F(3,60)=6.906, p<0.001^a^ | *** | 1 vs 2, p=0.9977, ns | p=0.0666 |
|  | |  |  | 1 vs 3, p=0.0249, * |  |
|  | |  |  | 1 vs 4, p=0.0013, ** |  |
| Figure S1A | | H=7.315, p=0.0625 | ns |  |  |
| Figure S1B | | H=4.01, p=0.260 | ns |  |  |
| Figure S1C | | F(3,53)=2.27, p=0.473 | ns |  | p=0.473 |
| Figure S1D | | F(3,28)=1.646, p=0.201 | ns |  | p=0.0926 |
| Figure S1E | | H=4.138, p=0.247 | ns |  |  |

^1^F(DFn, Dfd), p-value for one-way ANOVA and H, p-value for Kruskal-Wallis test. ^2^Significance level was set to 𝛼=0.05. ns indicates p≥0.05, * indicates p<0.05. ^3^Numbers correspond to boxplots from left to right. Adjusted p-values after Dunnett's multiple comparison or Dunn's multiple pairwise comparison test against the DMSO-treated control group. Significance level was set to 𝛼=0.05; ns p≥0.05, * p<0.05, ** p<0.01, *** p<0.001. ^4^Bartlett’s test for homogeneity of variances. Significance level was set to 𝛼=0.05.
